# Linking omentum and ovarian cancer: NO

**DOI:** 10.18632/oncoscience.248

**Published:** 2015-09-14

**Authors:** Bahar Salimian Rizi, Deepak Nagrath

**Affiliations:** Department of Chemical and Biomolecular Engineering, Rice University, Houston, TX, and Department of Bioengineering, Rice University, Houston, TX

**Keywords:** tumor microenvironment, cancer metabolism, nitric oxide, arginine metabolism, omentum

Ovarian cancers are one of the most lethal gynecological cancers because they are usually diagnosed in advanced stages when metastasis has already occurred in the peritoneal cavity. Interestingly, ovarian cancers frequently invade the fatty pad of adipose tissue along the stomach called the omentum [1]. Recent studies have shown that omentum contains a population of stem cell-like cells such as adipose stromal cells (ASCs) that engraft in tumors and encourage cancer progression [2]. Omentum-derived ASCs (O-ASCs) have been demonstrated to contribute to the formation of a hospitable environment for the development of ovarian cancer metastasis [2].

Previous studies have shown that O-ASCs enhance proliferation and migration of ovarian cancer cells while reducing their response to chemotherapy and radiation [2]. However, the mechanistic underpinnings of O-ASCs’ role in tumor progression and growth are still unclear. Recently we demonstrated that nitric oxide (NO)-mediated metabolic coupling between O-ASCs and ovarian cancer cells contributes to ovarian cancers’ growth and increased chemosensitivity [3]. Our study revealed that O-ASCs secreted arginine, which was used by cancer cells for NO generation. NO is generated endogenously by an enzymatic conversion of arginine into citrulline through nitric oxide synthase (NOS). NOS has been found to be differentially expressed in obese and non-obese individuals and has been shown to be overexpressed in aggressive ovarian tumors [4]. We revealed that O-ASCs secreted arginine could rescue reduced proliferation rates of ovarian cancers caused by arginine deprivation [3]. Notably, overweight O-ASCs were able to rescue the reduced proliferation of cancer cells more than their lean counterparts. However, more studies are required to shed light on the role of obesity in ovarian cancer development and progression.

Several studies indicate that NO is a double-edged molecule. It is a tumor promoter at lower concentrations (less than 500 nM), but damages DNA and induces apoptosis at higher concentrations (millimolar range) [5]. We found that ovarian cancer cells in cocultures with O-ASCs had significantly higher NO levels compared to their non-coculture counterparts. We previously showed that NO synthesis upregulates Warburg effect in ovarian cancers [5]. Interestingly, coculture of cancer cells with O-ASCs reduced oxygen consumption rates (OCR) in cancer cells. We confirmed this shift in ovarian cancers’ metabolism from oxidative phosphorylation (OXPHOS) to glycolysis by comparing the contributions of both pathways towards cellular ATP generation and our data elucidated that O-ASCs increased NO synthesis in cancer cells, resulting in suppression of mitochondrial respiration.

Previously, we showed that O-ASCs induced chemo-resistance in cancer cells [2]. Interestingly, in recent studies we found that O-ASCs-mediated chemoresistance can be deregulated by disrupting NO homeostasis [3]. We added L-arginase in direct-contact cocultures of O-ASCs and cancer cells. Both L-arginase (which depletes any secreted arginine by O-ASCs and thereby blocks NO synthesis in cancer cells) and L-NAME (a NO synthase inhibitor) in direct-contact cocultures of O-ASCs and ovarian cancer cells, increased chemosensitivity of paclitaxel in cancer cells. Our results suggest that combined approach of depleting arginine using L-arginase, along with inhibiting NO synthesis in cancer cells using L-NAME, may be a viable therapeutic approach for targeting ovarian cancers, to disrupt the communication between cancer cell and O-ASCs.

Arginine is not the only player in the metabolic coupling of O-ASCs and ovarian cancers. Surprisingly, O-ASCs-secreted arginine when is used for NO synthesis in cancer cells, generates citrulline as a byproduct, which is secreted by cancer cells and in turn is ingested by O-ASCs. We hypothesized that this high amount of secreted citrulline concentrations may be beneficial for NO metabolism in O-ASCs. As mentioned before, O-ASCs are multipotent population of mesenchymal stem cells that can differentiate into adipocytes. Surprisingly, we found that secreted citrulline by cancer cells significantly increased adipogenesis capacity of O-ASCs and stimulated the lipid droplet accumulation within O-ASCs. Studies have shown that NO inhibits lipolysis of lipid depots in subcutaneous adipose tissue [6]. This highlights the metabolic symbiosis between omentum-derived ASCs and cancer cells in maintaining NO homeostasis in tumors.

Future studies are needed to investigate the mechanism behind the modulation of ovarian tumor's metabolism by NO. The post-translational modification of metabolic enzymes caused by binding of NO with their cysteine residue (s-nitrosylation) may play a crucial role. S-nitrosylation has been known to alter the functions of enzymes in fatty acid metabolism as well as other central energy pathways [7]. The impact of obesity in ovarian cancer initiation and progression has been studied and further studies are needed to explore the role of NO metabolism in this modulation. Furthermore, the link between omentum and other hormonal tumors such prostate cancers needs to be further studied. Even as new mechanisms implicating omentum for ovarian cancer metastasis keep emerging, many aspect of its biology still remains to be unveiled [8].

**Figure 1:**
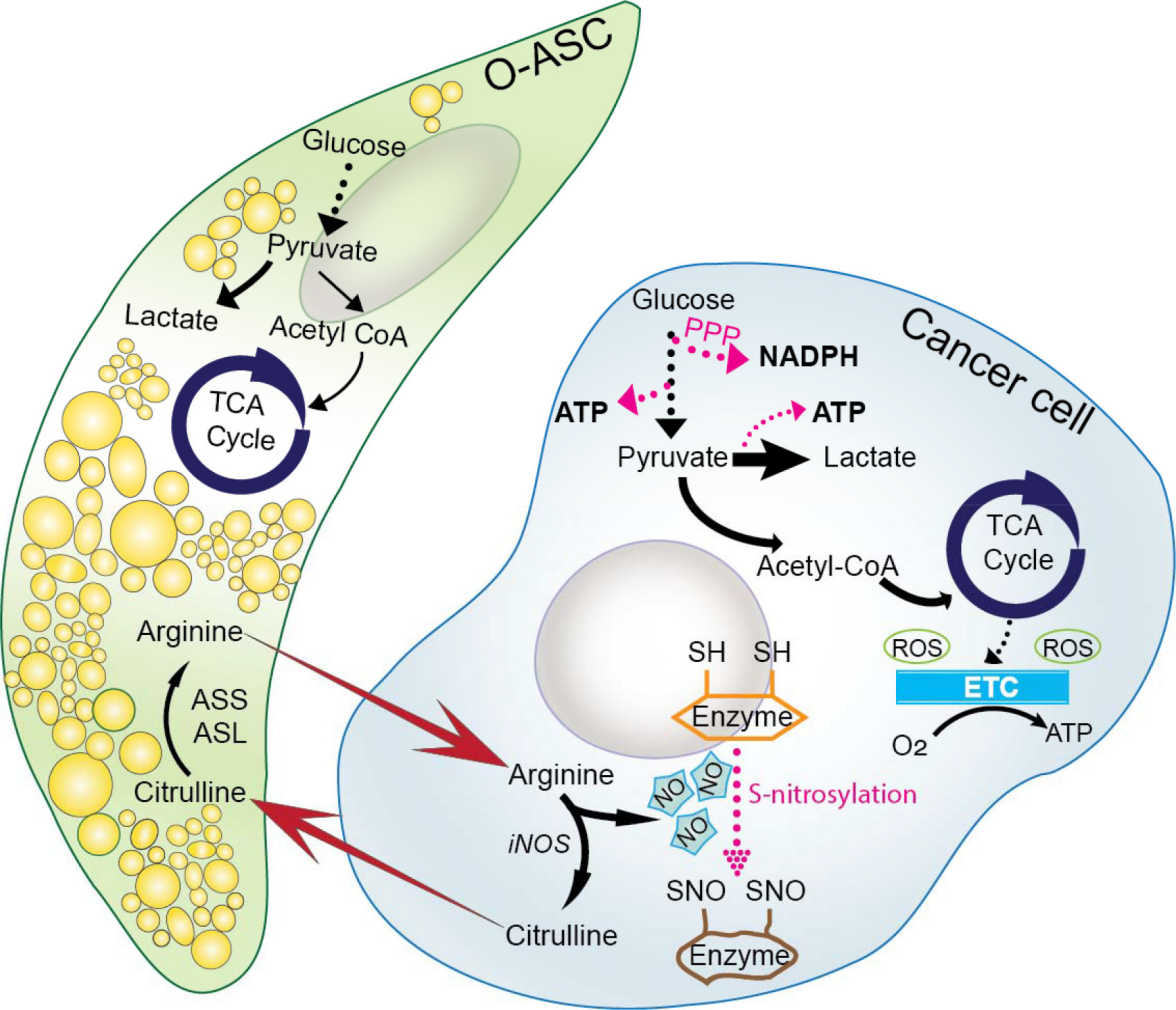
Crosstalk between ovarian cancers and O-ASCs in tumor microenvironment O-ASCs supply cancer cells with the pool of L-arginine for NO synthesis. The conversion of L-arginine into citrulline is through nitric oxide synthase (NOS). Moreover, secreted citrulline by cancer cells is consumed by O-ASCs and enhances adipogenicity of O-ASCs. The generated NO may induce s-nitrosylation of metabolic enzymes and may alter ovarian cancer cell metabolism.

## References

[1] Nieman KM (2013).

[2] Nowicka A (2013). PLoS One.

[3] Salimian Rizi B (2015). Cancer Res.

[4] Ryden M (2001). Int J Obes Relat Metab Disord.

[5] Caneba CA (2014). Cell Death Dis.

[6] Elizalde M (2000). J Lipid Res.

[7] Doulias PT (2013). Sci Signal.

[8] Cai J (2012). Carcinogenesis.

